# YouTube Doctors Confronting COVID-19: Scientific–Medical Dissemination on YouTube during the Outbreak of the Coronavirus Crisis

**DOI:** 10.3390/ijerph182111229

**Published:** 2021-10-26

**Authors:** Álex Buitrago, Alberto Martín-García

**Affiliations:** Department of Audio-Visual Communication and Advertising, Campus María Zambrano, University of Valladolid, Plaza de la Universidad 1, 40005 Segovia, Spain; alberto.martin.garcia@uva.es

**Keywords:** health communication, science communication, COVID-19, social media, YouTube, coronavirus

## Abstract

The coronavirus crisis has highlighted the consumption of social media and online video platforms in search of an alternative discourse to that provided by traditional media. The objective of this article is to find an approach to education and scientific dissemination on COVID-19 present in the Spanish context of YouTube, focusing on the content analysis of audiovisual texts generated by creators from the medical and biomedical fields. A methodological orientation was used for this based on the Grounded Theory, which was applied to five case studies whose informative material on SARS-CoV-2 exceeded ten million views as of March 2021. The results reveal the existence of a group of medical professionals in the Spanish YouTube sphere who, since the COVID-19 outbreak, have been involved in the construction of an alternative discourse around the health crisis and its evolution that pursues a high standard of audiovisual quality, scientific rigor, and educational ability.

## 1. Introduction

The COVID-19 crisis has caused substantial changes in citizen’s interaction with the media ecosystem [1,2]. Within the generalized increase in digital media consumption, the increase in users of online video streaming platforms stands out. On one hand, we find those that follow a unidirectional model (Netflix, Sky, HBO, etc.) who meet the standards of the so-called file-television: accessibility, mobility, and consumption on demand [3]. On the other hand, we find those who follow a multilateral network model. Among the latter, YouTube is undoubtedly the most popular social video platform with the highest following rates worldwide.

Somehow, we can say that the emergence of this health crisis accelerated (more than expected) the transition that was already in force from the broadcast model of traditional media to video-on-demand platforms. At the same time, the situation of information confusion generated by COVID-19 has caused a new intermedia collision [4], by showing the differences between consuming traditional media as the main source of information (newscasts, reference firms, press conferences of official institutions, experts selected by government bodies, etc.) or, preferably, resorting to agents of the new media ecosystem, such as those on which this research is focused: scientific-medical disseminators on YouTube. The premise on which this research is based on the following: The transformative contribution that YouTube is assuming as a vehicle for education and scientific dissemination on COVID-19 [5] is mirrored in the articulation of its own media texts, so an effective analysis should start with the study of media texts and progressively move towards studies that are more focused on technological processes. The choice of an approach that comprehensively addresses the phenomenon of science education and dissemination within the new media ecology added to a set of categories of semiotic-discursive analysis is ideal for the effective development of such an investigation. Likewise, the use of methodologies based on semiotics and discourse analysis has a long history in the study of audiovisual textuality, whether applied to the cinematographic narration [6,7,8,9], television story [10,11] or in empirical–cognitive approaches [12].

The present analysis of the Spanish disseminators on COVID-19 on YouTube should be considered as the presentation of a contextual map of a group of new educational and media agents with a specific communicative weight within the greatest global health crisis of the last hundred years. The textual corpus is made up of videos published by the five disseminators studied during the months of February–March–April 2020. When analyzing the production of these education-media agents, we start with the enunciation of three study questions:

− What kind of discursive strategies are using Spanish medical disseminators on COVID-19 on YouTube?
− What key lines of action are they proposing to users facing the COVID-19 crisis?
− What thematic and informative plots regarding the coronavirus can be identified in the production of Spanish medical disseminators focusing on COVID-19 on YouTube?

The first section of this article presents an introduction to the emerging phenomenon of academic, cultural, and scientific dissemination in the Spanish context of YouTube and justifies the suitability of media education as an appropriate theoretical framework for understanding the media emergence of this phenomenon and its mechanisms of adaptation. In the methodological section, the science communicators of YouTube are described as new education-media agents, and the main categories of the semiotic-discursive analysis implemented are displayed in a synthesized way. In addition, the eligibility criteria that the selected subjects had to meet to become part of the study corpus are established (that is, the requirements to be considered a legitimate medical disseminator on COVID-19 in the Spanish context of YouTube). The third section presents the analysis of the discourse of these science communicators, focusing on the taxonomy of their statements, genres, themes and enunciative contracts.

### The Emergence of a New Way of Cultural and Scientific Dissemination

Since its start in 2005 and its subsequent purchase by Google (now Alphabet Inc., Mountain View, CA, USA) for $1.65 billion, YouTube has been consolidating itself as a hegemonic online video platform with the highest levels of visibility [13,14]. However, and despite its obvious educational and training potentialities [15], it is usually associated with the field of social media entertainment (video games, humor, fashion/beauty, etc.) as its preeminent thematic sphere [16,17,18]. An extended belief about YouTube comes from the regular publication of rankings of the most-followed channels, which have always been dominated, including at the date of this study, by creators linked to gaming and entertainment. This vision may incur bias, and attempts to assign YouTube’s media discourse to only that of these authors, which would be like equating the global discourse of the cinematographic field to the one found exclusively in the movies with the highest attendance and revenue rates. This reduction overlooks the heterogeneity and expressive, discursive, thematic and aesthetic richness that inhabits both media realities.

According to Cunningham and Craig [16], there are three iconic genres of what is commonly known as ‘youtuber style’: videogames, beauty/DIY videos, and personality vlogs. These three kinds of video make up what these authors call “Social Media Entertainment” [17], a cultural conglomerate in which entertainment, advertising and digital platforms converge [19]. At the technical level, this ‘youtuber style’ uses medium shots, interspersed with close-ups. The camera usually remains static, although sometimes a handheld camera is used, with a normal height and angulation, or at a high angle. Regarding the editing, they opt for a fast pace with numerous cuts on the same take (jump cut), trying to avoid any silence or emptiness within the discourse (*horror vacui*).

The emergence of academic, cultural and scientific dissemination through online videos [20], and, in a singular way, on YouTube [21] has fostered research around a compendium of study focuses, highlighting: the phenomenon of *platformization* [22]; the discussion about YouTube as an element of participatory culture [23,24,25]; the popularity and distinctive features of its content creators, also known as Youtubers [26,27,28]; the key role of these Youtubers in the media consumption habits of children and adolescents [29,30,31,32,33]); immersion in specific types of cultural Youtubers, such as the so-called booktubers [19,34,35]. It is also worth mentioning studies referring to deliberations on the video format itself as an educational vehicle [36] for the dissemination of science [37], and the peculiarities of this format regarding scientific rigor [38], narrative possibilities [39], entertainment effect [40], production and audiovisual formats [41], etc.

One of the main disadvantages of disseminating science on YouTube is undoubtedly its coexistence on the same platform as misinformative or pseudoscientific channels. Non-scientific voices had a significant presence during the outbreak of the health crisis, coming from different profiles: openly pseudoscientific channels (e.g., Atraviesa lo desconocido), influencers on other topics who gave advice on COVID-19 (e.g., Paula Gonu), or channels that broadcast the statements of public personalities that spread hoaxes (e.g., presidents such as Donald Trump (USA), Andrés Manuel López Obrador (Mexico), Jail Bolsonaro (Brazil), etc.) Despite the existence of these channels, YouTube one of the largest audio–visual showcases at present, offering instant access to knowledge of all kinds and the democratization of global learning. However, there are also unflattering factors regarding the future of science communication on YouTube, despite the continuous increase in new dissemination channels opened by specialists and based on scientific evidence. The technological evolution of this platform occurs in the context of algorithmic control of content [42,43,44] which, added to external interference from political and economic powers [45,46,47], calls into question this *media-educational* utopia.

## 2. Materials and Methods

The methodological process began with an orientation based on the Grounded Theory [48] and materialized through the direct analysis of a selection of YouTube channels, whose creators had to meet the following seven eligibility criteria (previously established):

(1) Own a channel belonging to the Spanish context of YouTube and whose contents are developed in the Spanish language.
(2) Own a channel for scientific–medical dissemination; this purpose was to be explicitly stated in the Home or More Information sections of the Channel. Accepted terminology: Disclosure/Communication/Information, on Medicine/Medical Science/Scientific-Medical/Biomedical/Biomedicine/Biomedical Engineering.
(3) At least one year, in February 2020, since the publication of the first scientific-medical outreach video on their channel. The aim was to analyze medical disseminators with a solid track record on YouTube, avoiding creators who sprang up in the heat of the COVID-19 crisis without having previously published such work on the platform.
(4) A minimum of 50,000 subscribers.
(5) Careful video editing. The aim was to study content creators who carried out audio–visual dissemination with aesthetic and formal care, avoiding recordings of a single take without a minimal editing process.
(6) Individual characters (personal, private, independent) and non-institutional (company, collective, association, research centres, universities). As researchers in media studies, we were interested in the application of the Youtuber phenomenon to academic, cultural, and scientific dissemination, and not as interested in the casuistry of institutions that decided to open a YouTube channel as a complement to their work in other areas.
(7) Possess a higher degree (Bachelor’s degree or above) in Medicine or one of the following alternatives: Biomedicine, Biomedical Engineering.

Following these eligibility criteria, five YouTube channels were finally selected for the investigation (Table 1).

These channels, when validated as case studies, represent five accredited scientific-medical outreach projects in the context of YouTube in Spanish (a language with more than 500 million speakers throughout the world). All of them have a significant and quantifiable journey within the platform.

Despite its being the most recent channel, La Hiperactina (2018) has managed to position itself as a benchmark for biomedical outreach on YouTube and has achieved more than 130,000 subscribers in its first two years of existence. The other channels—Diario de un MIR (2007), Alberto Sanagustín (2011), Glóbulo azul (2011) and Iván Moreno (2017)—belong to four practising doctors who, since the beginning of the COVID-19 health crisis, have combined their professional tasks with outreach work on YouTube. La Hiperactina, Diario de un MIR and Glóbulo azul’s channels also belong to Cultube, a Spanish community/event of YouTube disseminators directly supported by the Ministry of Science and by Spanish Foundation for Science and Technology (FECYT). In addition, both Pau Mateo (Diario de un MIR) and Sandra Ortonobes (La Hiperactina) participated in the live broadcast of Science Truck: Science against the coronavirus (20 April 2020). Science Truck is FECYT’s project, aiming to combine several of the most prominent science YouTubers on the same channel, and is usually broadcast live from secondary schools. It is aimed at a young audience and has the objective of awakening interest in different scientific disciplines.

These elements, combined with their popularity as YouTubers (reflected in the number of subscribers and view count of their videos during the crisis), justifies the representative and referential nature of the selected sample.

Through a qualitative methodology, supported by grounded theory [48,49], a semiotic-discursive analysis was carried out of all the scientific–medical dissemination pieces collected in our corpus study (see Table A1 in Appendix A). The analytical process and its different phases were supported by the qualitative analysis suite Atlas.ti. The platform itself has served as a source for the collection of ethnographic research data.

### Coding and Categorization

To structure our analytical process, a set of semiotic-discursive categories was designed, and later applied to the study corpus:

− Expressive articulation: focused on the study of all those parameters linked to the enunciation of emotional, affective, or empathic elements by the disseminator, as well as their interweaving with the social reality generated by the COVID-19 crisis.
− Thematic taxonomy: focused on the study of all those matters concerning the COVID-19 health crisis and its classification into different thematic subcategories: pedagogy on the nature of the virus; symptomatology and treatment; prevention measures against COVID-19; global information and statistical data; experience as a health worker with direct treatments of the disease; etc.
− Interaction with the audience: focused on the study of all those direct and explicit appeals by the disseminator towards the user/consumer of the videos, whether they were verbal or visual, or linked to the theme of COVID-19, and regardless of the aim of the interaction (an invitation to interact, encouragement to act, etc.).

## 3. Results

The formal discourse of the five disseminators that were studied can be included -within the Youtuber aesthetic, in what has come to be called a *personality vlog* [16,19]. The camera monologue, confessional style, and the celebration of the *bedroom culture* [28] make up a discursive identity that, in the three selected channels, is contravened on very few occasions and for very justified reasons.

In reference to the assembly of the pieces, careful editing is perceived in the majority of them, very typical of the *personality vlog*, characterized by the continued use of the *jump cut*. Thanks to this type of transition, voids, breaths, fillers, etc., are suppressed without changing the frame, allowing the constant flow of verbal discourse, and reaffirming the *horror vacui* as one of the essential characteristics of the Youtuber style.

### 3.1. Expressive Articulation

Eleven study categories were previously established in the design of this research clan. Subsequently, three of them were deleted due to imprecision in defining them or similarity with other codes. In the next step, it was agreed to merge three of the remaining eight into one (appealing to the same area), thus forming the emotional reaction macro-category, which has six study subcategories. Therefore, analysis of the expressive articulation of the studied disseminators was established through 5 categories and 10 analytical subcategories (Table 2).

It is important to note that in 100% of the pieces studied we found the informative/didactic tone (first category in Table 2). The action of explaining a certain aspect, piece of data or concept regarding SARS-CoV-2 or COVID-19 to the camera was a common denominator within the study corpus. Emotional reactions were found in 77.26% of the videos. Among these, the use of an exclamatory tone was most present (18.18%) of all the studied emotions. The adoption of a critical tone also acquired a specific weight in the disseminators’ discourse. Specifically, 59.09% of the videos uttered criticisms or complaints about any of the four following analysis subcategories. The most criticized area was *citizen behaviour* (22.72%), followed by criticism *of someone in particular* (18.18%). Among those collected in the sample, disapproval of the Instagram influencer Paula Gonu (@paulagonu) (2 million followers), Andrés Manuel López Obrador (President of Mexico) and Olivier Véran (Minister of Health of France) stands out. These expressions of disapproval were all made in relation to erroneous or unscientific information provided by the individuals. Although its weight was reduced, it is worth mentioning the presence of self-criticism (9.09%) in the discourse of the disseminators. Thus, even though they were specialists in the medical or biomedical field, the five creators repeatedly stressed that the information available about SARS-CoV-2 was fickle and, therefore, the content of their videos could become obsolete overnight. In fact, Diario de un MIR dedicated a complete video to apologizing for the information provided in one of its previous publications: “Os pido mis sinceras disculpas [I sincerely apologize to you]” (13 March 2020).

As a last remarkable piece of information, in 40.9% of the analyzed material, the disseminators resorted to humor (comments of hilarious tone/laughter/comedy/affability) as a way to brighten the story about COVID-19 that was addressed in each video.

A significant level of recurrence was not observed in the remaining categories and subcategories, and the adoption of an ironic/sarcastic tone was the one with the lowest frequency of appearance (4.54%).

### 3.2. Thematic Taxonomy

With 103 coded citations, this research group achieved the greatest quantitative weight within the study. Initially, the material was codified on seven analytical categories, with four new categories added during the experimental work (direct viewing of the pieces). Finally, during the accountancy process, it was decided to merge two codes into one (for reasons of similarity), so the thematic taxonomy was fixed as 10 categories and 24 analytical subcategories (Table 3).

**Figure 1:**
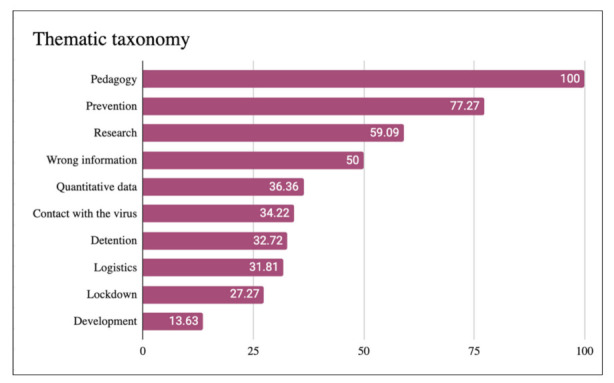
Thematic taxonomy in percentages.

The category that reached the greatest recurrence was the pedagogical one (Figure 1). In this way, we can affirm that in 100% of the studied pieces, a pedagogical discourse was shared on one of the three subcategories collected by the study. Specifically, in 45.45% of cases, pedagogy was carried out regarding the virus itself (SARS-CoV-2) and the disease it causes (COVID-19); 13.63% of discourse was on the origin/history of coronaviruses, and 40.90% of discourse focused on aspects related to virology and bioscience in general.

The second theme to reach the highest significance quota, present in 77.27% of the study corpus, was the one referring to *prevention* measures and tools. In third place we find the category dedicated to *research* (vaccine/scientific evidence/authority citations), with a mention percentage of 59.09%. Finally, in exactly half of the informative pieces studied (50%), various pieces of information circulating in the media were pointed out as erroneous. In other words, they tried to dismantle widespread hoaxes and label certain content as misinformative or pseudoscientific. Recurrence exceeded 36% in none of the remaining categories, with the disease development theme having the lowest frequency of appearance (13.63%).

**Figure 2:**
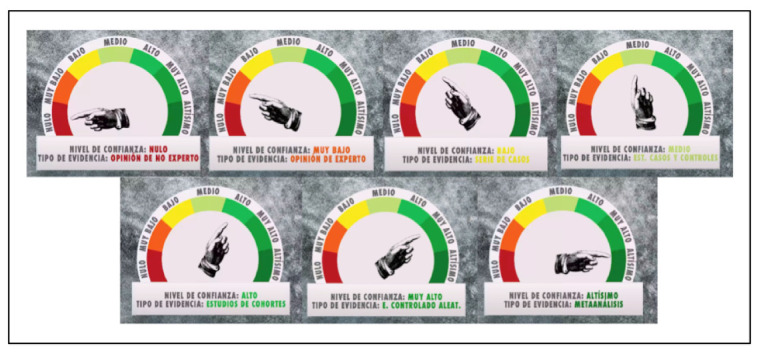
Marker of scientific evidence from Diario de un MIR.

Alone, the pedagogical function has the greatest presence in the speeches of Glóbulo Azul and La Hiperactina, and the category of prevention measures and tools was most discussed by Diario de un MIR. In the case of the latter disseminator, it is worth highlighting the creation of a marker of scientific evidence that provides a validity rating to each piece of information/discovery about COVID-19 mentioned on the channel. There are seven possible ratings, called *confidence levels*, with each one associated with a certain *type of scientific evidence*. Confidence levels (the type of associated evidence in parentheses) were as follows: (i) highest (meta-analysis), (ii) very high (randomized controlled study), (iii) high (cohort studies), (iv) medium (case-control study), (v) low (case series), (vi) very low (expert opinion), (vii) null (non-expert opinion). The attribution of a level of trust is visually manifested through a logo, which appears simultaneously with the verbal transmission of new information or discovery. In the logo, the seven ranks are listed by colour, together with a marker that indicates the assigned level (Figure 2).

### 3.3. Interaction with the Audience

To study this research group, seven initial categories of analysis were designed, which were later expanded to nine (after a direct viewing of the pieces during the experimental phase). Later, in the accountancy process, we decided to merge four of the previously established categories (for reasons of similarity) under the *non-thematic appeal* label. Finally, the study of interaction with the audience was fixed through 6 categories and 15 analytical subcategories (Table 4).

**Figure 3:**
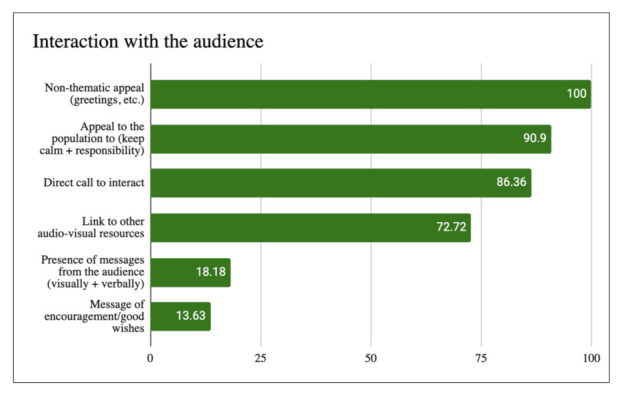
Interaction with the audience in percentages.

The category with the most significant presence within the sample was the so-called *appeal to the population*, which appeared in 90.9% of the studied cases (Figure 3). Appeals to remain calm and not fall into a state of collective hysteria due to the expansion of the pandemic occurred in 27.27% of the cases. A total of 63.63% of the cases were calls for the population to be responsible and to not trivialize of the health crisis generated by COVID-19. However, in the most significant data extracted from the analysis of this category, in our opinion, the last call for calm within the sample was recorded on 16 March 2020, in the video “COVID-19 ¿Por qué estamos en estado de alarma? (COVID-19 Why are we in a state of alarm?)” of *Glóbulo Azul*. From that moment on, only *calls for responsibility* were registered. We consider that, as 3 months of material are covered in this study (February–March–April), the fact that, following the middle of the study, the *appeals to calm* disappear and only repeated calls to not trivialize COVID-19 are registered is considerably significant. In the case of Diario de un MIR, the last call for calm dated from 24 February 2020, although it should be noted that Pau Mateo (the channel’s owner and disseminator) was working during this period, studying as an emergency doctor in Italy (the main focus of the virus in Europe) in the city of Piacenza (a municipality bordering the Lombardy region, the epicentre of the infection). Likewise, Pau Mateo recognizes in his video “Os pido mis sinceras disculpas (I ask you for my sincere apologies)” (13 March 2020) that a video published on 26 February, in which he intended to convey calmness, was deleted because “the message that you perceived or that seems to be sent was completely wrong”.

In the second category with the highest percentage of appearance, we find a discursive element typical of the Youtuber style outside the scope of COVID-19: the direct invitation to interact (86.36%). Within this category, the four established subcategories (like it/send comment/share video/subscription) obtain very even results: close to 20% include an invitation to leave a comment, with this being the most present subcategory of the four (22.72%). It is worth mentioning that the number of comments received on a video is closely linked to the positioning of that video in the YouTube search engine and the number of appearances it receives in the user recommendation system, as both mechanisms are based on algorithmic neural networks. Therefore, the greater the number of messages received, the better the video’s positioning and the more the video appears in the YouTube recommendation system. However, despite this moderate request to insert comments (which are mainly found in Diario de un MIR’s videos), the appearance of messages from the audience in the videos (either visually or verbally) was not reflected to the same extent (18.18%), with visual mentions prioritized over verbal mentions. Therefore, users did not play a relevant role in the construction of the discourse in the studied cases.

Third, the *links to other audiovisual resources* had a significant presence, appearing in 72.72% of the cases studied. Among them, the main option was to direct the user to view other videos from the same channel (42.18%), either previously published videos or future pieces that the creator announces will be released in the following days/weeks.

No significant level of recurrence was observed in the remaining categories and subcategories, with the transmission of *messages of encouragement/good wishes* appearing least frequently (13.63%).

Regarding the number of views the studied pieces received, a significant decreasing pattern in the number of views per video is perceived as time progresses within the period of study (February–March–April 2020), that is, as the COVID-19 health crisis progressed. The most obvious example of this pattern was found in Glóbulo Azul, since, with a single exception, his new videos always lost viewers with respect to the previously published piece. In this way, among the nine pieces analyzed in the study corpus, the first of them (“Real doctor explains the coronavirus (COVID-19)” (2 March 2020)) registered 243,899 views (consulted on 20 May 2020), while the last of the videos included in the sample (“Mental health in times of COVID-19” (26 April 2020)) obtained 10,444 views (consulted on 20 May 2020). Meanwhile, La Hiperactina practically halved its number of viewers from the first to the second of the two videos that form part of the corpus. Specifically, 164,774 views were registered for “Coronavirus, What happened?” (6 February 2020) and 98,197 for “Coronavirus Update” (26 March 2020) (accessed on 20 May 2020). Diario de un MIR presented somewhat more variable viewing data, with various audience peaks reached throughout its 11 videos studied. However, these occasional increases could be explained by the deliberate inclusion of certain elements in the title of the piece, such as the mention of being in the epicenter of the virus in Europe, direct appeals to the president of another country (with the consequent attraction of viewers of that nationality), or direct appeals to the health alarm. In this way, the viewer peaks reached by Diario de un MIR, listed chronologically and computed as of May 2020, are as follows: 774,250 for “Message from a doctor at ground zero of the coronavirus in Italy” (24 February 2020); 451,265 for “Message from an emergency doctor in Italy to the president of Mexico” (24 March 2020); 537,352 for “If we continue like this we are going to have a strong 2nd wave (I hope I’m wrong)” (10 April 2020). Beyond these three specific peaks, a progressive decrease can be seen in the number of viewers of Diario de un MIR’s other pieces as time progresses.

## 4. Discussion

The results of the research inform us of the existence of a group of professionals in the medical and biomedical fields who, after the emergence of COVID-19, became involved in the construction of an alternative discourse around the health crisis and its evolution that pursues a high standard of visual quality, scientific rigor, and educational ability. This online audio–visual education and dissemination effort adds value to the global media flow in difficult times, distancing itself from the official narrative, which prevails in much of the conventional media (TV, radio, and general press).

In addition, these professionals carried out their informative work in a medium in which fake news and misinformation abound. In this sense, not only does the internet foster the proliferation of fake news, it also has a polarized and uncritical social character beyond the digital environment. This polarization is largely promoted by social media due to the existence of information bubbles, that is, environments produced by algorithms exclusively displaying content related to the user’s beliefs and affinities. Therefore, there could be a domino effect: the internet fosters the polarization and uncritical nature of society, which favors the proliferation of fake news. Thus, misinformation is encouraged, and truthful scientific content is downplayed.

Regarding the mechanisms of influence, the main elements of persuasion, which are very present in YouTube discourse, refer to the sender of the message, the content of the message, how the message is communicated and the audience [50]. In most cases, the audience pays more attention to the characteristics of the sender than the context of the message or its content [51], and this is usually true for YouTube influencers, since any information they offer will likely be believed by their followers. Therefore, it is important that these science influencers exist, as they base their content on objective facts and scientific evidence, and not on beliefs (even if they use traditional persuasive mechanisms).

Regarding the expressive articulation of the message by the disseminators, the informative tone always prevailed when any aspects concerning COVID-19 were addressed in each video. However, this didactic, expressive modality was not an obstacle to the appearance of emotional reactions in most of the pieces. The emotional burden linked to the dawn of the health crisis and its initial development (February–March–April) made the almost permanent coexistence of informative and emotional expressiveness inevitable in each publication. Criticism of certain institutional and civic attitudes was also not lacking in most of the studied pieces. These criticisms were made in a constructive way and from a professional medical approach, always pointing out the appropriate procedure to correct any reprehensible attitude.

The collected thematic taxonomy revealed a wide range of plots focused on the coronavirus crisis, prioritizing pedagogical work, which could be found in all the studied pieces. Beyond dissertations on the description and operation of SARS-CoV-19 and COVID-19, the most recurrent themes in disseminators’ discourse were prevention measures and tools, and the scope of research around COVID-19. Regarding this last plot, disseminators’ insistence on indicating whether or not there was scientific evidence in their pieces is significant. Some even went so far as to design their own marker of scientific evidence, as in the case of Diario de un MIR. Likewise, we note that, in half of their videos, the scientific–medical disseminators of YouTube took advantage of their position and dismantled hoaxes, pointed out widespread misinformation, or directly qualified certain content as misinformative or pseudoscientific.

The interaction with the audience was primarily characterized by constant direct appeals to the user/viewer, either appealing to the audience to remain calm and not to fall into hysteria or calling for responsibility and asking the audience to not trivialize the looming health crisis. However, the most significant trend was found when observing how calls for calm gradually gave way to calls for responsibility, with no call for calm found beyond 16 March 2020 (the midpoint of the study period). Therefore, it is observed that, among the disseminators, initial calmness and skepticism were gradually transformed into concern, alertness, and awareness of the magnitude of the virus.

Alongside interactions regarding COVID-19, the high level of direct invitations to the users to interact with the pieces reveals that the authors did not lose sight of their identity as creators of YouTube content whose visibility and impact directly depends on their figures, view counts, comments, subscriptions, etc. Therefore, it appears that the scientific–medical dissemination vocation of these youtubers coexists with their desire to progress on the platform, seeking user interaction so that the algorithmic neural networks that regulate YouTube promote their content as much as possible.

However, this constant request for feedback from the audience was not equally reflected a posteriori in the disseminators’ successive audiovisual texts, since readings or mentions of messages from users had a remarkably reduced presence. Therefore, the construction of disseminators’ scientific–medical discourse on YouTube follows a unidirectional dynamic and has a limited relevance to the user.

Regarding the quantitative study of the views, a decreasing pattern can be perceived in the numbers of views per video as the period studied progressed. This trend could be explained by various factors: (1) the progressive loss of the freshness factor after the outbreak of the health crisis; (2) the information saturation on COVID-19 that the audience experienced as confinement progressed; (3) the gradual reduction in aspects, themes or approaches not already covered in previous videos.

## 5. Conclusions

In short, we consider this study to be one of the first approaches to the production of scientific–medical disseminators on COVID-19 in the Spanish context of YouTube, thus highlighting a research aspect that expressly links the audiovisual texts of emerging digital platforms with medical disclosure during the outbreak of the coronavirus health crisis.

Future research, therefore, should comprise a comparative analysis with scientific–medical YouTubers from other geographical areas, countries, and languages, analyzing their similarities and differences. Even within the Spanish language, it would be of interest to focus on other nations that were notoriously affected by COVID-19, such as Peru, Chile or Mexico.

Regarding the limitations, we should mention that we could have included a larger sample of channels or a longer analysis period (longer than three months). However, our aim was to analyze science communication during the “irruption” of the COVID-19 crisis, when truthful and contrasting information on COVID-19 was still meagre and insufficient. It could also be interesting to broaden the studied dissemination spectrum beyond the medical angle and verify the approach that, within YouTube itself, other scientific disseminators specializing in physics, chemistry, biology, or biotechnology, among others, are offering information on the health crisis.

Finally, apart from YouTube’s hegemony, signs of scientific dissemination on COVID-19 have been detected in other emerging platforms based on live-streaming, such as Twitch.tv. Different vehicles and new formats could pose suggestive challenges for researchers focusing on media studies.

We firmly believe that the online audio–visual dissemination on COVID-19, when based on scientific rigor and evidence, is an example of the real and effective application of media education development in society amid the greatest transnational health crisis of the last century. We are aware of the commercial impediments that a platform such as YouTube, in the hands of Google (Alphabet Inc., Mountain View, CA, USA), poses for the implementation of a truly horizontal and participatory culture.

The neural networks that control its recommendations system, the incremental context of algorithmic censorship and the decreasing monetization of content for its authors are aspects that threaten the techno-utopian vision of an open, free, massive, and democratic virtual platform where academic, cultural, and scientific dissemination flow without barriers and contribute to citizen empowerment within the digital media ecosystem. Despite this, we consider that YouTube, as the COVID-19 crisis has shown, continues to be inhabited by channels that represent good practices and provide stimulating experiences within the dissemination and educational field. Therefore, researchers in media studies should pay attention to such creators, as well as an enriching and lasting dialogue should be established between both groups. 

## Figures and Tables

**Table 1:** YouTube channels included in the study corpus.

Channel	Creator	Degree	Number of Subscribers (March 2021)	COVID-19 Videos (2/3/4—2020)	Accumulated Views (March 2021)
Glóbulo azul	Amyad Raduán	Medicine	235,000	9	1,006,218
Diario de un MIR	Pau Mateo	Medicine	102,600	11	2,299,419
La Hiperactina	Sandra Ortonobes	Biomedicine	130,000	2	262,971
Iván Moreno	Iván Moreno	Medicine	78,800	17	2,208,951
Alberto Sanagustín	Alberto Sanagustín	Medicine	447,000	15	4,735,080

**Table 2:** Codebook for the analysis of expressive articulation.

1. Informative/Didactic Tone	
2. Emotional reaction	2.1. Exclamation *(including bad language)*
	2.2. Emotionality
	2.3. Despair/grief
	2.4. Empathy
	2.5. Annoyance
	2.6. Fear
3. Criticism/complaint about:	3.1. Institutional measures
	3.2. Citizen behaviour
	3.3. Someone in particular
	3.4. Self-criticism
4. Hilarious escape/laughter/affability	
5. Irony/sarcasm	

**Table 3:** Codebook for Thematic Taxonomy Analysis.

1. Pedagogy	1.1. SARS-CoV-2/COVID-19
	1.2. Origin/History of coronaviruses
	1.3. Virology/Bioscience in general
2. Detention	2.1. Symptomatology
	2.2. Diagnosis/Test
3. Contact with the virus	3.1. Infection
	3.2. Transmission
4. Development	4.1. Treatment
	4.2. Pharmacology
5. Prevention	5.1. Preventive tools
	5.2. Recommendations for action
6. Lockdown	6.1. Tips for confinement–quarantine
	6.2. Psychological consequences
7. Wrong information	7.1. Hoaxes
	7.2. Misinformation
	7.3. Pseudoscience
8. Quantitative data	8.1. Transnational data
	8.2. Statistical information/Ratios
9. Logistics	9.1. Hospitals
	9.2. Healthcare system
	9.3. Own experience as a professional
10. Research	10.1. Vaccine
	10.2. Scientific evidence (studies, papers)
	10.3. Authority quotes

**Table 4:** Codebook for the analysis of the interaction with the audience.

1. Non-thematic appeal	1.1. Presentation
	1.2. Initial greeting
	1.3. Farewell
	1.4. Gratefulness
2. Message of encouragement/good wishes	
3. Appeal to the population to	3.1. Keep calm/keep tranquil/no hysteria
	3.2. Responsibility/non-trivialization
4. Presence of messages from the audience	4.1. Verbally
	4.2. Visually
5. Link to other audiovisual resources	5.1. Other videos from the same channel (past and future)
	5.2. Other networks of the disseminator
	5.3. Other creators
6. Direct call to interact	6.1. Like it
	6.2. Send comment
	6.3. Share video
	6.4. Channel subscription

## Data Availability

Data sharing is not applicable to this article.

## Appendix A

**Table A1:** Characteristics of the 54 videos included in the analysis.

YouTube Channel	Video Name	Date	Visualizations	Comments	Duration
Glóbulo azul	MEDICO REAL EXPLICA EL CORONAVIRUS (COVID-19) Causas, Sintomas, Diagnostico, Tratamiento, Patologia https://bit.ly/3DeQTm7	2 March 2020	246,505	994	9:39
Glóbulo azul	3 médicos responden al ministerio https://bit.ly/3mw48YO	7 March 2020	133,608	403	3:15
Glóbulo azul	Médico responde a Paula Gonu sobre el Coronavirus https://bit.ly/2WLhveS	13 March 2020	249,238	1067	25:21
Glóbulo azul	¿Por qué estamos en estado de alarma? ¿Cuánto de grave es la situación? https://bit.ly/3FmowUZ	16 March 2020	120,426	517	7:42
Glóbulo azul	¿El Ibuprofeno empeora la enfermedad por Coronavirus? https://bit.ly/3FldeQS	16 March 2020	112,577	442	7:22
Glóbulo azul	¿La cura contra el Coronavirus (COVID-19)? ¿Y la vacuna? https://bit.ly/2Ykym8M	21 March 2020	62,087	250	8:13
Glóbulo azul	¿Cómo parar el Coronovarius/COVID-19? Esta es la estrategia https://bit.ly/3uNaffh	25 March 2020	46,552	264	10:36
Glóbulo azul	¿Fin de la cuarentena? https://bit.ly/2YpiUrP	10 April 2020	48,724	382	5:00
Glóbulo azul	Salud mental en tiempos de COVID-19 https://bit.ly/2ZWhMNf	26 April 2020	13,161	93	5:44
Diario de un MIR	¿Cómo puedo evitar infectarme de Coronavirus? https://bit.ly/3AaIY7q	3 February 2020	73,884	463	18:04
Diario de un MIR	Las nuevas medidas que se toman en mi hospital https://bit.ly/3oAbtJF	25 February 2020	131,237	851	7:30
Diario de un MIR	España: Aprended de lo que pasa en Italia, por favor https://bit.ly/3msmRo8	12 March 2020	61,059	610	2:29
Diario de un MIR	Os pido mis sinceras disculpas https://bit.ly/3le0qDK	13 March 2020	168,847	2.135	33:39
Diario de un MIR	¿Podéis confiar en Diario de un MIR? https://bit.ly/3uJFpUN	20 March 2020	36,578	212	9:24
Diario de un MIR	Mensaje de un médico de urgencias en Italia al presidente de México https://bit.ly/3AaJO44	24 March 2020	456,779	2968	5:23
Diario de un MIR	19 consejos para hacer un confinamiento de pacientes en casa https://bit.ly/3ad3SIy	24 March 2020	29,030	167	6:14
Diario de un MIR	¿En qué consiste el test para el Coronavirus? https://bit.ly/2WKBYQX	26 March 2020	24,615	147	10:15
Diario de un MIR	¡Nueva investigación importante! ¿Diagnóstico precoz de COVID-19? https://bit.ly/3FiBNxX	5 April 2020	40,288	179	12:45
Diario de un MIR	Si seguimos así vamos a tener una segunda oleada fuerte (espero equivocarme) https://bit.ly/3ixjviD	10 April 2020	542,211	3521	7:30
Diario de un MIR	¡Que no te pase esto! Casi me desmayo en mi primera cirugía. https://bit.ly/3mqPRgc	12 April 2020	32,817	182	11:00
La Hiperactina	Coronavirus, ¿qué ha pasado? https://bit.ly/2YynX9Q	6 March 2020	217,938	1057	13:14
La Hiperactina	Actualización sobre el Coronavirus https://bit.ly/3DfRZ0P	26 March 2020	153,010	1163	15:39
Iván Moreno	Presentación y situación general COVID-19 https://bit.ly/3mtMFQP	17 March 2020	8418	11	12:54
Iván Moreno	Actualización Covid 18 marzo https://bit.ly/3a9KlbN	18 March 2020	1556	0	10:01
Iván Moreno	Actualización Covid del 20 de marzo https://bit.ly/3iC20gY	20 March 2020	1791	5	15:19
Iván Moreno	Actualización Covid del 22 de marzo https://bit.ly/3iBEGQA	22 March 2020	1497	2	11:24
Iván Moreno	Creo que tengo Coronavirus, ¿qué hago? https://bit.ly/3mqt0RT	23 March 2020	11,259	6	12:11
Iván Moreno	Actualización Covid del 24 de marzo https://bit.ly/302MaFV	24 March 2020	2301	1	13:21
Iván Moreno	Coronavirus, ¿cómo salimos de esta? (1 de 3) https://bit.ly/3msxAiz	27 March 2020	18,044	9	12:09
Iván Moreno	Coronavirus, ¿cómo salimos de esta? (2 de 3) https://bit.ly/3lerfrm	27 March 2020	9156	0	13:48
Iván Moreno	Coronavirus, ¿cómo salimos de esta? (3 de 3) https://bit.ly/2Ywk3OX	27 March 2020	12,623	14	9:38
Iván Moreno	Introducción nuevo formato RCP COVID-19 https://bit.ly/3AaQjnD	14 April 2020	10,115	28	7:34
Iván Moreno	De guardia https://bit.ly/3Bgf17l	15 April 2020	895,420	390	9:34
Iván Moreno	Sobre los test en Covid (1 de 2: tipos y usos) https://bit.ly/2ZW9rsV	17 April 2020	100,578	77	13:59
Iván Moreno	Sobre los test en Covid (2 de 2: interés individual y poblacional) https://bit.ly/3uK6fft	17 April 2020	51,217	169	18:30
Iván Moreno	Confianza ¿ciega? https://bit.ly/3iDNnd3	20 April 2020	44,916	171	17:39
Iván Moreno	Antihipertensivos y COVID-19 https://bit.ly/3uMWQnf	21 April 2020	35,023	45	5:43
Iván Moreno	Lo que aporta una semana… https://bit.ly/3AgkbPe	25 April 2020	931,429	732	19:30
Iván Moreno	Especial preguntas y respuestas https://bit.ly/2WJBURs	27 April 2020	97,124	369	1:51:06
Alberto Sanagustín	El Coronavirus ya está aquí (COVID-19) https://bit.ly/3DhsufS	29 February 2020	44,960	97	16:29
Alberto Sanagustín	La verdad sobre el Coronavirus (reflexiones de un médico de familia) https://bit.ly/3uJSH3o	7 March 2020	999,532	1770	24:59
Alberto Sanagustín	Coronavirus. Qué está pasando? (Reflexiones de un médico de familia) https://bit.ly/3BkAD2y	14 March 2020	135,555	591	12:19
Alberto Sanagustín	Spiriman, Sálvame y el Coronavirus (médico de familia de Ibiza le responde) https://bit.ly/2YqLTeQ	18 March 2020	1,080,576	2585	19:59
Alberto Sanagustín	Coronavirus: miserables y canallas (testimonios profesionales) https://bit.ly/3oGYeXx	21 March 2020	1,271,797	2857	8:35
Alberto Sanagustín	Coronavirus: alarmismo, sanitarios contagiados, confinamiento y testimonios https://bit.ly/3uLK5cx	23 March 2020	167,558	1317	22:29
Alberto Sanagustín	Coronavirus: políticos y Spiriman (donaciones) https://bit.ly/3oKtZii	24 March 2020	107,278	1014	21:11
Alberto Sanagustín	Casos reales, atención primaria y cloroquina/hidroxicloroquina. https://bit.ly/3uNbPgQ	28 March 2020	179,439	692	10:56
Alberto Sanagustín	Coronavius: lo bueno, lo feo y lo malo https://bit.ly/3ldjbY3	4 April 2020	105,354	561	28:13
Alberto Sanagustín	Test de Roth: dificultad respiratoria https://bit.ly/3lfTxSl	5 April 2020	230,001	471	4:42
Alberto Sanagustín	Cómo protegerse del Coronavirus SARS Cov-2 https://bit.ly/3uTpP9a	11 April 2020	112,733	512	19:05
Alberto Sanagustín	Desconfinamiento: ¿nuevo pico? https://bit.ly/3leBbBa	15 April 2020	117,795	492	13:47
Alberto Sanagustín	¿Te pondrás la vacuna del Coronavirus cuando esté disponible? https://bit.ly/3AijmW6	21 April 2020	292,279	1231	4:03
Alberto Sanagustín	Desconfinamientos, brotes en otoño y ¿culpables de la pandemia? https://bit.ly/3oClTZ7	25 April 2020	74,972	575	22:42
Alberto Sanagustín	¡Me timaron! (Mascarillas y material sanitario) https://bit.ly/3uJm1qV	26 April 2020	43,725	611	7:34

All included links were last accessed on 20 September 2021.
